# Humanized mice reveal an essential role for human hepatocytes in the development of the liver immune system

**DOI:** 10.1038/s41419-018-0720-9

**Published:** 2018-06-04

**Authors:** Jinglong Guo, Yang Li, Yanhong Shan, Chang Shu, Feng Wang, Xue Wang, Ge Zheng, Jin He, Zheng Hu, Yong-Guang Yang

**Affiliations:** 1 Institute of Translational Medicine, The First Hospital, Jilin University, 130061 Changchun, China; 2 National-Local Joint Engineering Laboratory of Animal Models for Human Diseases, 130061 Changchun, China; 3 International Center of Future Science, Jilin University, 130012 Changchun, China; 4 Columbia Center for Translational Immunology, Department of Medicine, Columbia University College of Physicians and Surgeons, New York, NY 10032 USA; 5 Hepatopancreatobiliary Surgery Department, The Second Hospital, Jilin University, 130041 Changchun, China

## Abstract

The liver is an immunological organ with a distinct immune cell profile. Although the composition and function of liver immune cells have been widely investigated, the mechanisms regulating the development and homeostasis of the specialized immune system, especially in humans, remain largely unknown. Herein, we address this question in humanized mice (hu-mice) that were constructed by transplantation of human fetal thymus and CD34^+^ hematopoietic stem/progenitor cells in immunodeficient mice with or without autologous human hepatocyte engraftment. Although the levels of human immune cell reconstitution in peripheral blood and spleen were comparable between hu-mice with and without human hepatocyte engraftment, the former group showed that human immune cell reconstitution in the liver was significantly improved. Notably, human immune cells, including Kupffer cells, dendritic cells and natural killer cells, were shown to be closely colocalized with human hepatocytes in the liver. Human hepatocytes engrafted in the mouse liver were found to produce IL-3, IL-15, GM-CSF, M-CSF, MCP-1, CXCL-1 and CXCL-10, which are known to be important for immune cell development, differentiation, tissue migration and retention, and have no or poor cross-reaction between humans and mice. Furthermore, human hepatocytes were able to support human immune cell survival and expansion in an in vitro co-culture assay. This study demonstrates an essential role for hepatocytes in the development and maintenance of the liver immune cell profile. The hu-mouse model with human autologous immune cell and hepatocyte reconstitution has potential for use in studies of the pathogenesis of liver immune disorders such as hepatotropic virus infections.

## Introduction

The liver is an important organ consisting of a large number and unique populations of immune cells. Compared with other tissues, the liver is populated with a high proportion of innate immune cells including natural killer (NK) cells, NK-like T cells, Kupffer cells and dendritic cells (DCs), which play important roles in local immune surveillance, liver regeneration and the pathogenesis of liver diseases^1–4^. The liver is well known as an immune tolerogenic organ^5–7^, but also develops rapid and vigorous immune responses under certain conditions, such as virus-induced fulminant hepatitis^8–10^. Previous studies have shown that hepatocytes are important for the immune tolerogenic properties of the liver^11–14^. Animal studies have shown that hepatocyte transplantation inhibits allograft rejection^15^, and hepatocytes have been reported to induce interleukin-10 (IL-10)-producing CD4 T cells through upregulation of Jagged1, a ligand of Notch signaling on T cells^14^.

Parenchymal tissue cells are thought to play an important role in creating a tissue microenvironment favoring recruitment, survival and self-renewal of the corresponding tissue-specific immune cell populations, that is, tissue-resident immune cells such as macrophages, NK cells and T cells^16–20^. A recent report showed improved development of human immune cells in human CD34^+^ cell-transplanted immunodeficient fah^-/-^ mice with human hepatoblast engraftment, indicating a potential stimulatory effect of hepatocytes on immune cell reconstitution^21^. However, the precise role of hepatocytes in the development and maintenance of the liver-specific immune system remains largely unknown. We have previously shown that immunodeficient mice receiving co-transplantation of human fetal thymic tissue (FTHY) and CD34^+^ hematopoietic stem/progenitor cells (HSPCs) develop a robust human immune system^22,23^. Although these humanized mice (hu-mice; also known as BLT hu-mice^24^) have been demonstrated to develop functional human immune cells and secondary lymphoid organs, and widely used to assess human immune responses in vivo under physiological or diseased conditions^25,26^, their tissue-specific immune reconstitution has not been explored well. In this study, we observed that human immune reconstitution in the liver was markedly improved in hu-mice that had been grafted with human hepatocytes, and that human immune cells in the liver were located mainly in areas enriched with human hepatocyte clusters. The engrafted human hepatocytes were found to produce a number of cytokines essential for lympho-hematopoiesis and chemokines involved in immune cell migration and tissue retention. Furthermore, co-culture experiments demonstrated that human hepatocytes can support the survival and proliferation of human immune cells. Taken together, this study provides not only a useful protocol for constructing hu-mice with improved liver-specific immunity, but also direct evidence for an important role for hepatocytes in the development of the specialized human immune system in the liver.

## Results

### Human hepatocyte repopulation in the liver of Jo2 antibody-treated immunodeficient mice

**Fig. 1:**
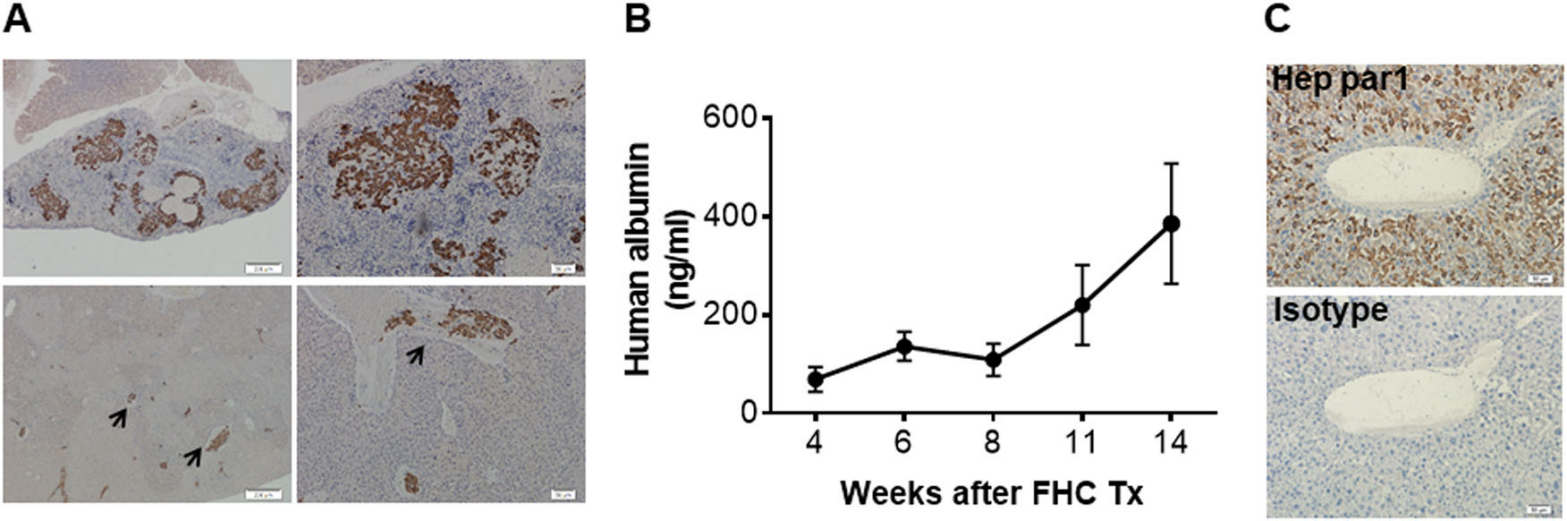
Human hepatocyte repopulation in the liver of Jo2 antibody-treated immunodeficient mice. NCG mice received intrasplenic transplantation of human FHCs, followed next day by Jo2 antibody treatment (0.3 mg/kg per injection every 3 days; i.p.) for 10 weeks. **a** Representative images of IHC staining with anti-human CK8/18 antibody on spleen (top) and liver (bottom) tissues harvested from of NCG mice 24 h after transplantation. Scale bars on the left and right panels are 200 μm and 50 μm, respectively. **b** Serum levels (mean ± SEMs) of human albumin in NCG mice that received FHC transplantation and Jo2 antibody treatment at the indicated time points (*n* = 15, 15, 12, 9 and 12 for 3, 6, 8, 11 and 14 weeks, respectively). **c** Representative images of IHC staining with anti-human Hep par1 (top) and isotype control antibody (mouse IgG1κ; bottom) of liver tissues from a representative mouse 6 weeks after FHC transplantation. Scale bars represent 50 μm

Human hepatocyte repopulation in the mouse liver was achieved by transplantation of human fetal hepatocytes (FHCs) followed by treatment with anti-Fas antibody (Jo2), which has been shown to induce mouse, but not human, hepatocyte apoptosis^27,28^. Briefly, NCG mice received an intrasplenic injection of FHCs, followed one day later by intraperitoneal injection of Jo2 at 0.3 mg/kg per injection every 3 days, a dose and schedule that was confirmed to be safe and effective (Figure S1). In these mice, migration of FHCs from the spleen to the port vein of the liver was observed as early as 24 h after transplantation (Fig. 1a). Human albumin was detected in the serum, with its level gradually increasing over time, indicating that functional human hepatocytes were successfully engrafted in these mice (Fig. 1b). Immunohistochemical (IHC) staining of mouse liver sections with anti-human Hep par1 antibody further confirmed the presence of living human hepatocytes in the liver (Fig. 1c). Accordingly, human-specific transcripts of hepatocyte genes such as *ALB* (albumin) and *AFP* (alpha-fetoprotein) were detected in liver tissues from these mice (Figure S2A). We also analyzed the expression of human hepatocyte-specific metabolic genes, including phase I enzymes *CYP1A2* (cytochrome P450 1A2) and *CYP2D6* (cytochrome P450 2D6), phase II enzyme *UGT1A1* (bilirubin UDP-glucuronosyltransferase 1–1), and transporters *MRP2* (multi-drug resistance-associated protein 2) and *NTCP* (Na^+^/taurocholate cotransporting polypeptide), in the liver tissues of the reconstituted mice compared with adult and fetal human liver tissues by quantitative reverse transcriptase-PCR (qRT-PCR) (Figure S2B). The levels of these metabolic gene transcripts (normalized to human *GAPDH*) in the chimeric mouse liver, although not reaching the levels attained in adult human liver, were greater than those in human fetal liver (Figure S2B), indicating a continued differentiation and maturation of FHCs in the mouse recipients. Collectively, these data demonstrate that the Jo2 antibody-based conditioning regimen allows successful and durable engraftment of functional human hepatocytes in immunodeficient mice.

### Human immune reconstitution in hu-mice with or without human hepatocyte engraftment

**Fig. 2:**
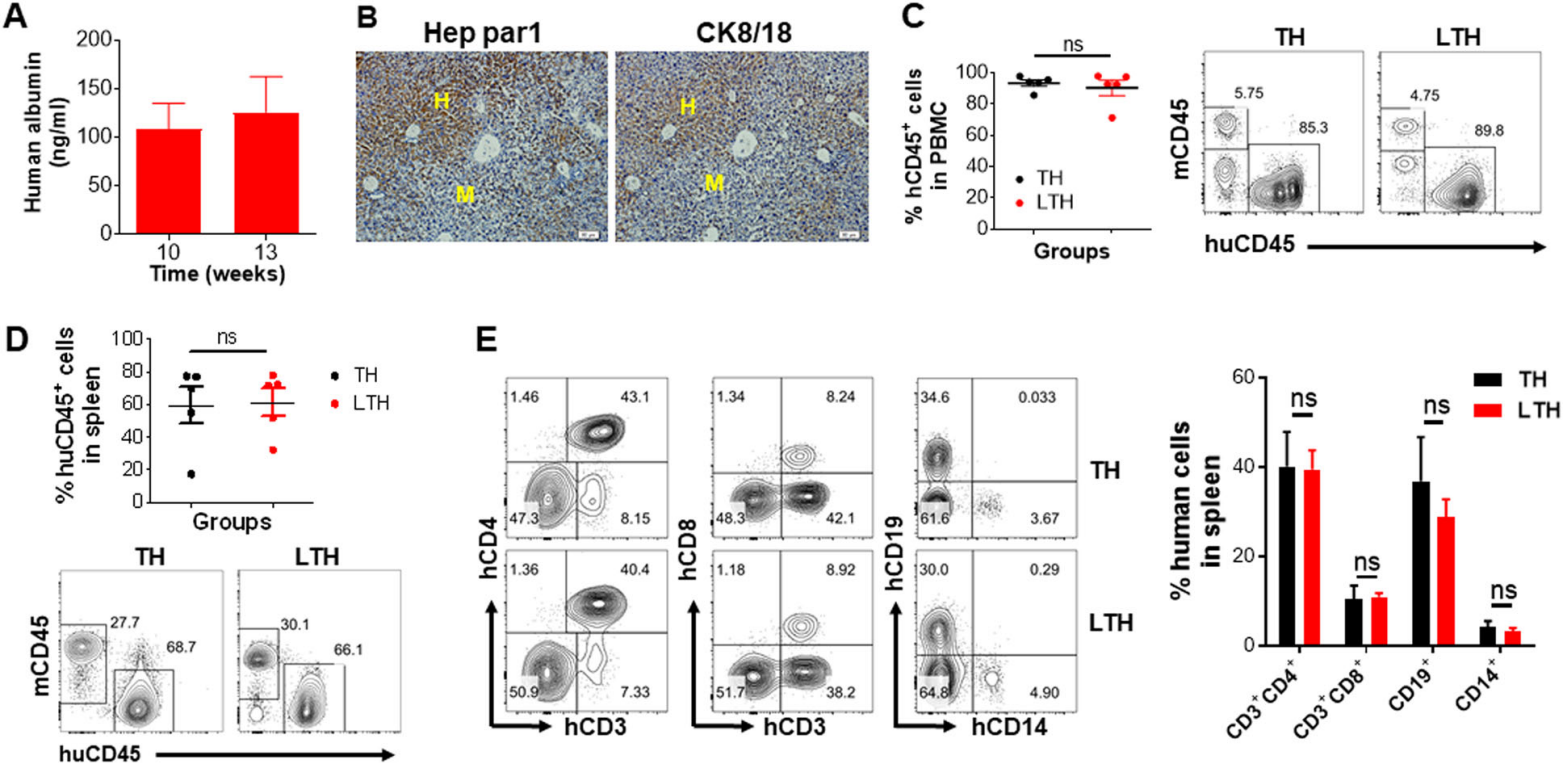
Human lymphohematopoietic chimerism in TH and LTH hu-mice. **a** Human albumin level in the serum of LTH hu-mice at indicated time point after human FHC transplantation (*n* = 10; mean ± SEMs). **b** Immunohistochemically staining with anti-human Hep par1 (left) and anti-human CK8/18 (right) antibodies of liver tissues from LTH hu-mice 15 weeks after human FHC transplantation. H human hepatocytes, M mouse hepatocytes. Scale bar, 50 μm. **c**-**e** FCM analysis for the levels of human CD45^+^ lymphohematopoietic cells in PBMCs **c** and spleens **d**, and percentages of human CD3^+^CD4^+^ and CD3^+^CD8^+^ T cells, CD19^+^ B cells, and CD14^+^ monocytes/macrophages in the spleens **e** of TH hu-mice and LTH hu-mice at week 15 post-transplantation of human FTHY and CD34^+^ HSPCs. Data are presented as mean ± SEMs (*n* = 5 per group)

We next attempted to establish an autologous human immune system in human hepatocyte-repopulated NCG mice by transplantation of human FTHY graft (under the renal capsule) and CD34^+^ HSPCs (intravenously) from the same fetus. As elucidated in Figure S3, NCG mice were first transplanted with FHCs and after 3 weeks were conditioned by sublethal irradiation, followed within the same day by the transplantation of human FTHY and CD34^+^ HSPCs (referred to as LTH hu-mice). All mice were treated with Jo2 (0.3 mg/kg every 3 days; intraperitoneally) starting 1 day after the transplantation of human hepatocytes. Again, all these mice showed the successful engraftment of human hepatocytes, as demonstrated by the production of human albumin (Fig. 2a) and the presence of human Hep par1^+^ and CK8/18^+^ hepatocytes (Fig. 2b). The average level of FHC repopulation in the liver of these mice, as measured by human Hep par1^+^ cells on IHC, was about 23% (Figure S4). These mice also showed high levels of human lymphohematopoietic cell chimerism in blood (Fig. 2c) and spleen (Fig. 2d), including CD3^+^CD4^+^ and CD3^+^CD8^+^ T cells, CD19^+^ B cells and CD14^+^ myeloid cells (Fig. 2e). However, human hepatocyte engraftment in the liver seemed to have no significant impact on human lymphohematopoietic reconstitution, as the levels of human CD45^+^, CD3^+^CD4^+^, CD3^+^CD8^+^, CD19^+^ and CD14^+^ cells in the LTH hu-mice were comparable to those in TH hu-mice (i.e., hu-mice transplanted with human FTHY and CD34^+ ^HSPCs only; Fig. 2c-e). IHC analysis revealed no significant difference in the numbers of human CD68^+^, CD11c^+^ or CD94^+^ cells in the spleens between LTH and TH hu-mice (Figure S5).

**Fig. 3:**
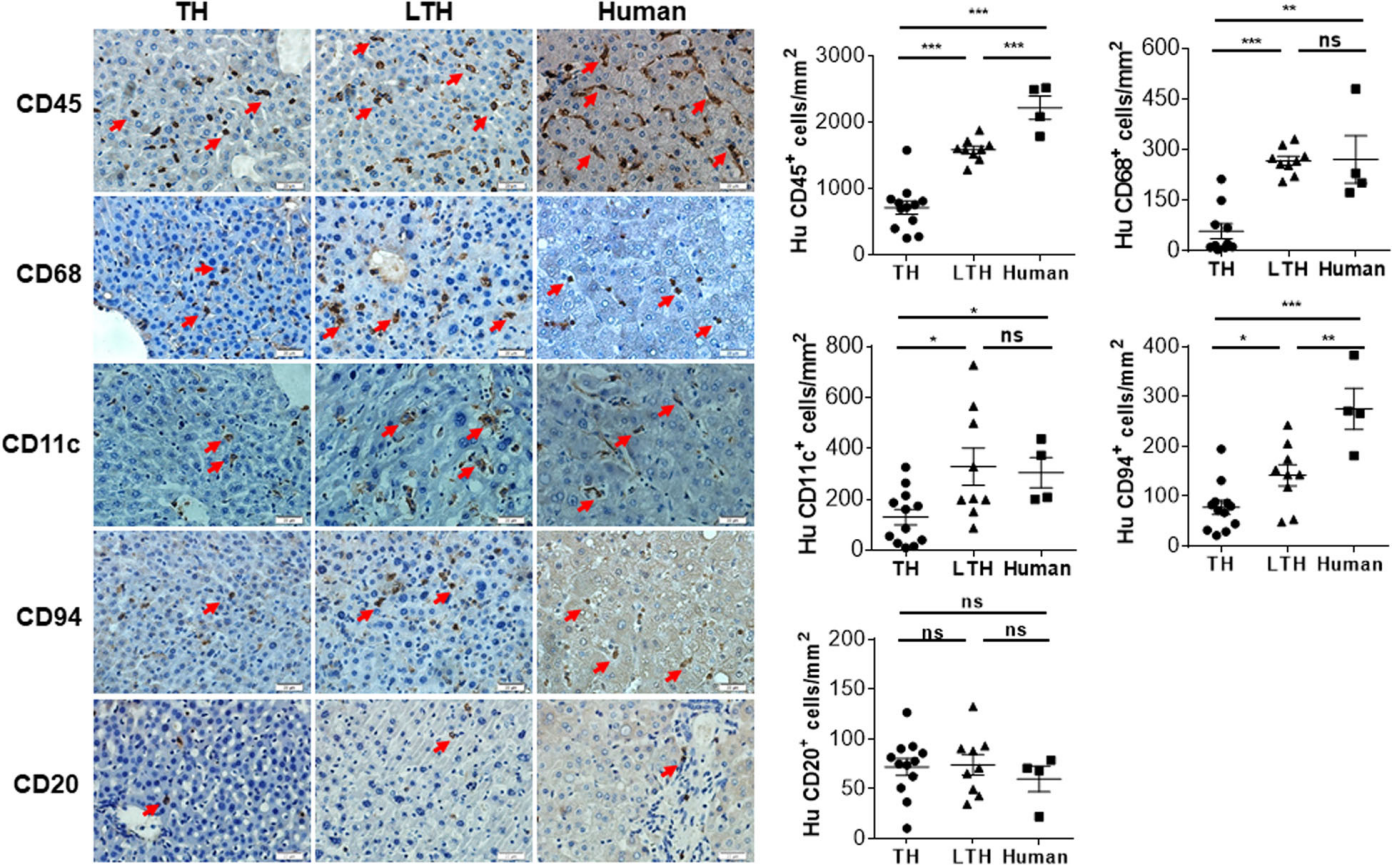
Human immune cell reconstitution in the liver of TH and LTH hu-mice. Liver tissues from TH hu-mice (*n* = 12; 14–20 weeks after transplantation), LTH hu-mice (*n* = 9; around 15 weeks after transplantation) and adult human donors (*n* = 4; aged 38–66 years) were immunohistochemically stained with anti-human CD45, CD68, CD11c, CD94 and CD20. Shown are representative photos (left) and densities of positively stained cells (right; mean ± SEMs). *p < 0.05; **p < 0.005; ***p < 0.001; n.s. not significant (unpaired, two-tailed Student’s *t*-test)

We next performed histological analysis to determine whether or not human hepatocyte engraftment can alter human immune cell reconstitution locally in the liver. Surprisingly, although the density of human CD45^+^ cells in the liver tissues from LTH hu-mice did not reach the level of adult human liver tissues, it was significantly higher than that in TH hu-mice (Fig. 3). With the exception of human CD20^+^ B cells, the densities of all other lineages of human immune cells examined, including CD68^+^ (macrophages or Kupffer cells), CD11c^+^ (likely DCs), and CD94^+^ (NK cells) cells in the liver samples from LTH hu-mice were significantly greater than in those from TH hu-mice. Furthermore, the densities of human CD68^+^ and CD11c^+^ cells in liver tissues of LTH hu-mice were comparable to those in adult liver samples (Fig. 3). These data indicate that human hepatocyte engraftment significantly promotes human immune cell reconstitution in the liver.

### Colocalization of human immune cells with engrafted human hepatocytes in LTH hu-mice

**Fig. 4:**
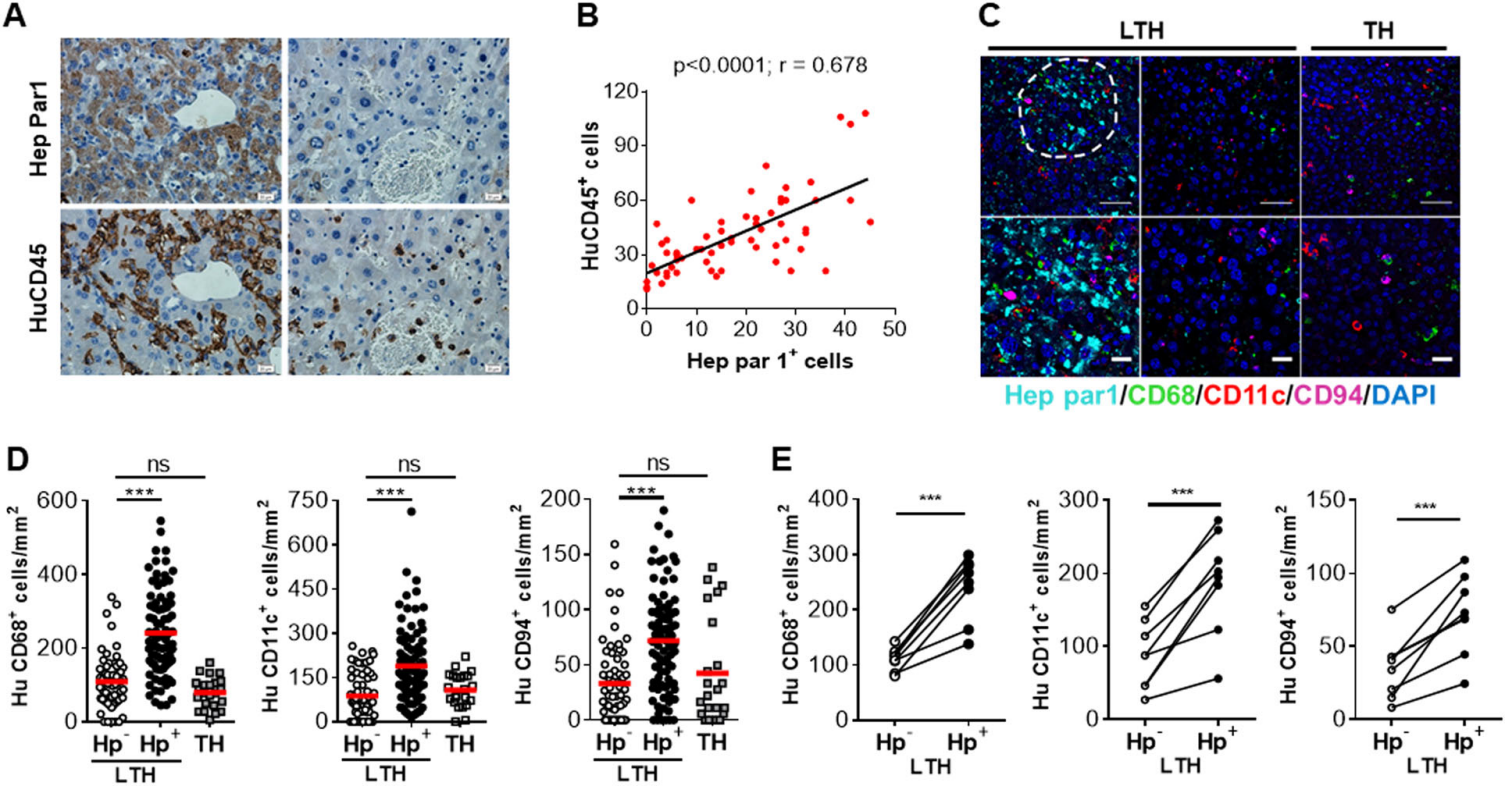
Colocalization of human immune cells with human hepatocytes in the liver of LTH hu-mice. **a,** **b** Serial sections of liver tissues from LTH hu-mice at approximately 15 weeks after transplantation of human FTHY/CD34^+^ HSPCs (*n* = 8) were stained with anti-human Hep par1 or CD45 antibodies. **a** Representative IHC staining images for microscopic areas enriched with (left panel) and lacked (right panel) human Hep par1^+^ cells (scale bar, 20 μm). Sections stained with anti-human Hep par1 and anti-human CD45 are shown in the top and bottom panels, respectively. **b** Pearson correlation between Hep par1^+^ and CD45^+^ cell numbers (measured in the original magnification × 200 photos). Correlation is significant at the 0.01 level (two-tailed), *p* < 0.0001, *R* = 0.678. **c**-**g** Liver tissues from LTH hu-mice (*n* = 8) and TH hu-mice (*n* = 2) were analyzed by multiplexed IHC assays using anti-human Hep par1 (cyan)/ CD68 (green)/CD11c (red)/CD94 (magenta) and DAPI (blue). **c** Representative staining images of liver tissues from LTH hu-mice (left and right panels show the microscopic areas enriched with and the areas lacking human Hep par1^+^ hepatocytes, respectively) and TH hu-mice were shown. Scale bars represent 50 μm (top) and 20 μm (bottom), respectively. **d** The densities (per mm^2^) of human CD68^+^, CD11c^+^ and CD94^+^ cells in the microscopic areas lacked (Hp^−^) or enriched with (Hp^+^) human Hep par1^+^ hepatocytes of liver tissues from LTH hu-mice, and the randomly selected areas in the liver tissues from TH hu-mice. Each symbol represents the density of positively stained cells in each individual microscopic area (10–12 areas were counted for each liver sample, and data are analyzed using unpaired, two-tailed Student’s *t*-test). **e** Densities of human CD68^+^, CD11c^+^ and CD94^+^ cells in the Hep par1^−^ (Hp^−^) and Hep par1^+^ (Hp^+^) areas of liver tissues from LTH hu-mice. Data shown are the average densities of positively stained cells in the Hep par1^-^ (Hp^-^) and Hep par1^+^ (Hp^+^) areas of each individual liver tissue (values from the same mouse liver tissue are connected with a line), and analyzed using paired, two-tailed Student’s *t*-test. ***p < 0.001; n.s. not significant

To further understand the role of human hepatocytes in human immune cell reconstitution in the liver, we assessed the spatial association between human immune cells and hepatocytes in LTH hu-mice. IHC staining of consecutive liver sections with anti-human Hep par1 and CD45 revealed that human CD45^+^ cells were present mainly in areas enriched with human Hep par1^+^ hepatocyte clusters (Fig. 4a), and there was a strong correlation between the density of human Hep par1^+^ and CD45^+^ cells (*p* < 0.0001; Fig. 4b). Multiplexed IHC staining further confirmed the colocalization of human Hep par1^+^ hepatocytes with human CD68^+^, CD11c^+^ and CD94^+^ cells. The densities of human CD68^+^, CD11c^+^ and CD94^+^ cells in the areas enriched with human Hep par1^+^ hepatocytes were significantly higher than in the areas lacking human Hep par1^+^ hepatocytes in liver tissues from LTH hu-mice (Fig. 4c-e). Furthermore, the densities of human CD68^+^, CD11c^+^ and CD94^+^ cells in areas lacking human Hep par1^+^ cells in liver sections of LTH hu-mice were comparable to those in liver tissues from TH hu-mice (Fig. 4c-e). Collectively, these data suggest that the interaction of human immune cells with human hepatocytes, either directly or indirectly through cytokine production, plays an important role in recruiting and/or maintaining human immune cells in the liver, that is, in the development of the liver-specific immune cell network.

### Human hepatocytes promote human immune cell survival and/or expansion in vitro

**Fig. 5:**
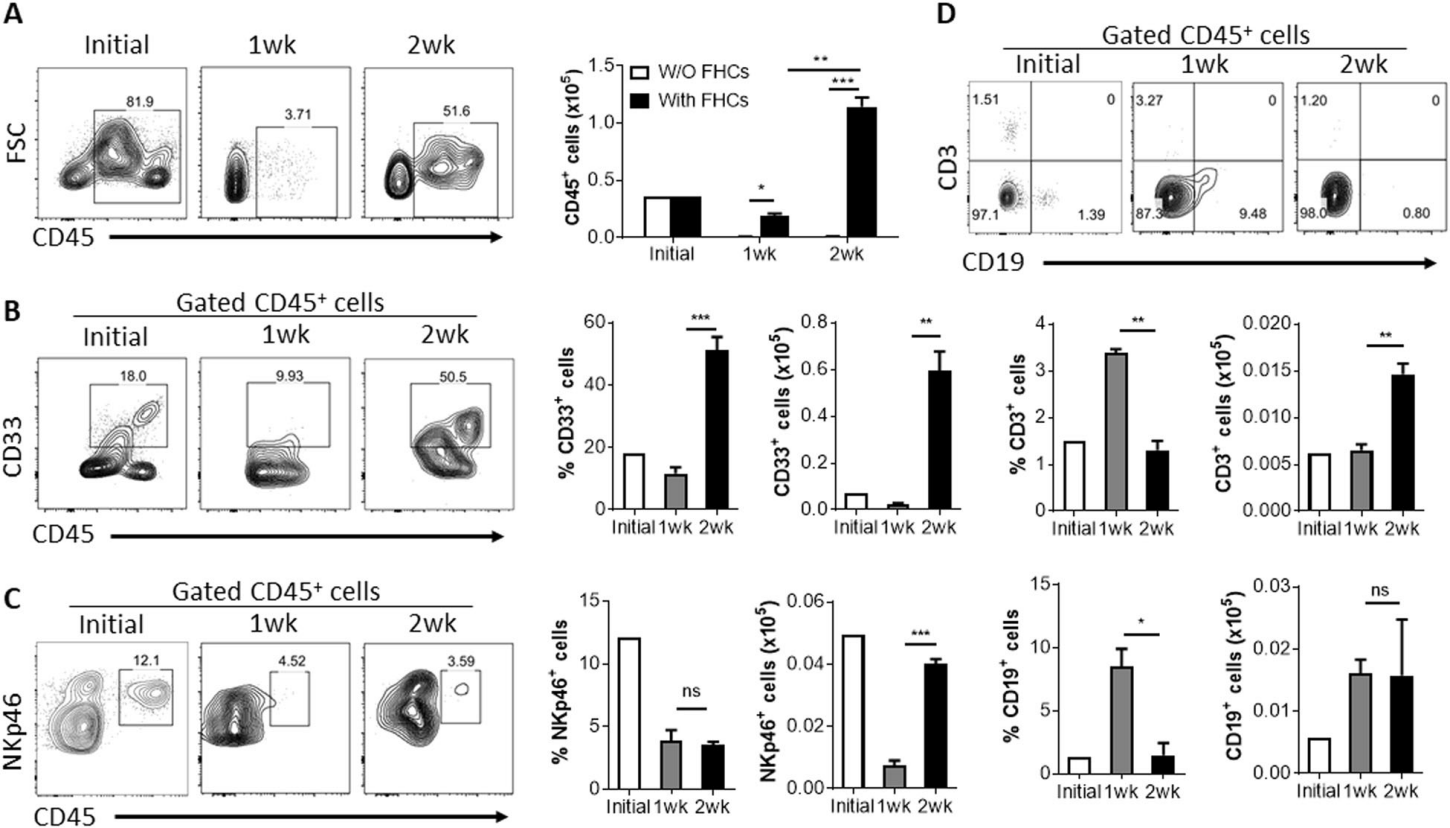
Human hepatocytes promote multiple lineage human immune cell proliferation in vitro. Human CD45^+^ cells purified from fetal liver were cultured in vitro at 5 × 10^4^/well in rat-tail collagen-coated 24-well plate with or without FHCs (2 × 10^5^ per well). **a** Numbers of CD45^+^ cell added (counted immediately before placed into culture plate; Initial), 1 week and 2 weeks after culture. **b**-**d** Percentages and numbers of CD33^+^ cells **b**, NKp46^+^ cells **c** and CD3^+^ and CD19^+^ cells **d** in the initial CD45^+^ cell population and the cell populations harvested 1 week and 2 weeks after cultured with FHCs. Data are presented as mean ± SEMs (*n* = 3). *p < 0.05, **p < 0.01, ***p < 0.001

To determine the potential of human hepatocytes for supporting the survival and/or expansion of human immune cells, we cultured human CD45^+^ cells, which were isolated from the CD34^−^ fraction of fetal liver cells (FLCs) following MACS purification of CD34^+^ cells, in the presence or absence of autologous FHCs (with a purity of >90%, Figure S6) and cellularity was determined after culture for 1 and 2 weeks. In the absence of human FHCs, almost all CD45^+^ cells died within a week (Fig. 5a). In contrast, the number of CD45^+^ cells expanded more than threefold in 2 weeks in the presence of human FHCs (Fig. 5a). Moreover, the initial CD45^+^ cell population isolated from fetal liver was comprised of human CD33^+^ myeloid, NKp46^+^ NK, CD3^+^ T, and CD19^+^ B cells, which were all detected and even expanded at the end of the 2-week culture (Fig. 5b-d). These data provide direct evidence that human hepatocytes can support the survival and proliferation of human immune cells, at least under in vitro conditions.

### Production of cytokines and chemokines by engrafted human hepatocytes in mice

**Fig. 6:**
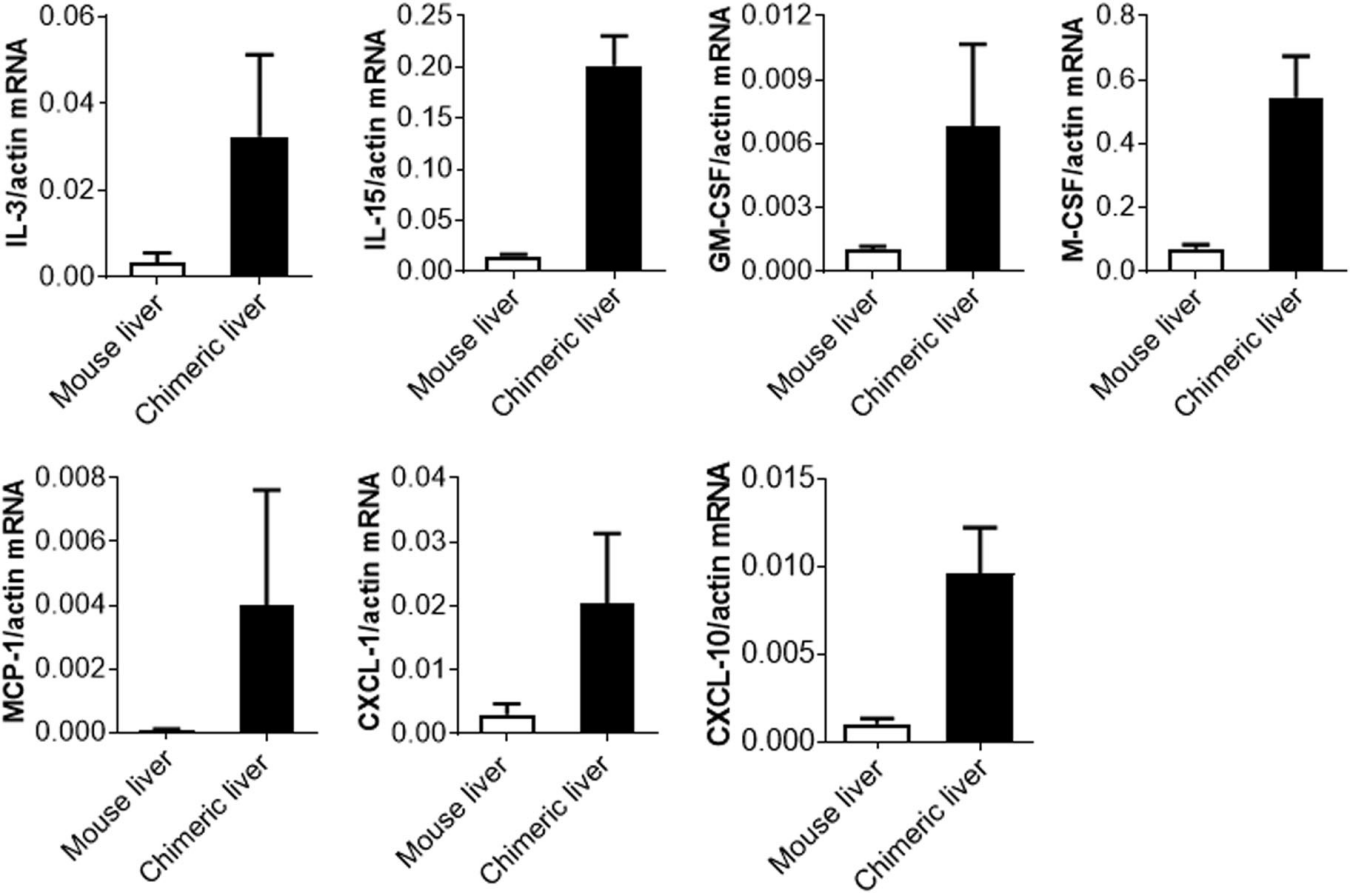
Human cytokine and chemokine gene expression in liver tissues from NCG mice that were grafted with human FHCs. Expression levels of human *IL-3, IL-15, GM-CSF, M-CSF*, *MCP-1*, *CXCL-1* and *CXCL-10* in liver tissues from human FHC-grafted NCG mice (chimeric liver; *n* = 3; harvested 14 weeks after FHC transplantation) or control NCG mice (*n* = 3). Shown are qRT-PCR results that are normalized to mouse plus human *ACTIN*

To further understand the role of engrafted human hepatocytes in the development and maintenance of the human immune system in the mouse liver, liver tissues were harvested from human hepatocyte-grafted mice and analyzed for the gene expression of human hematopoietic cytokines and chemokines, including cytokines that are essential for hematopoiesis and myeloid differentiation and maturation such as IL-3^29^, GM-CSF (Granulocyte-macrophage colony-stimulating factor)^30^ and M-CSF (Macrophage colony-stimulating factor)^31^, an important T and NK cell growth factor IL-15^32^, and chemokines that are important for immune cell tissue homing or retention such as MCP-1 (Monocyte chemoattractant protein 1)^33^, CXCL-1 (The chemokine (C-X-C motif) ligand 1)^34^ and CXCL-10^35^. As shown in Fig. 6, the expression of mRNAs for all these human cytokines and chemokines was clearly detected in liver tissues from mice with human hepatocyte engraftment but not in the non-transplanted control mice. The lack of or inefficient cross-reactivity of human cells to mouse IL-3^36^, IL-15^37^, GM-CSF^38^ and M-CSF^25,39,40^ is considered an important factor causing the failure of functional human NK cell development and the relatively poor reconstitution with human myeloid cells in TH hu-mice^25^. Given the low amino-acid identity between mouse and human MCP-1 (68.4%), CXCL-1 (57.6%) and CXCL-10 (68.4%)^25^, it is possible that human immune cells also exhibit reduced tissue-homing potential in mice. Together, these results indicate that human cytokine/chemokine production by the engrafted human hepatocytes is one of the important factors leading to improved reconstitution of the human immune system in the liver of LTH hu-mice.

## Discussion

Emerging evidence indicates that tissue-resident immune cells are key players in local immunity and immunopathology, and are essential for tissue function and homeostasis^17,18^. Although the mechanisms remain poorly understood, the composition of tissue-resident immune cells is known to differ among tissues^17,18^. Normally, the liver is populated with a high proportion of innate immune cells, including NK cells, NK-like T cells, Kupffer cells and DCs, forming an liver-specific immune network that is important in liver regeneration, local immune surveillance and immunopathogenesis^1–4^. In this study, we could observe that human hepatocyte engraftment significantly improves human immune cell reconstitution in the liver of hu-mice. Although the levels of human immune cell reconstitution in blood and spleen were comparable between TH and LTH hu-mice, the latter exhibited significantly improved reconstitution with human immune cells, including macrophages, DCs and NK cells, in the liver. Importantly, human immune cells were found to be colocalized in the liver with human hepatocytes, suggesting that the constant interaction of human immune cells with human hepatocytes or human hepatocyte-derived soluble factors may play an important role in the development and/or maintenance of the human immune system in the liver. Supporting this possibility, we found that human hepatocytes can promote the differentiation and expansion of human myeloid cells, NK cells and T cells in an in vitro assay.

Tissue cells produce chemokines that are important in regulating the migration and/or accumulation of immune cells in the tissue^41,42^. Previous studies have shown that hepatocytes produce a number of chemokines, such as MCP-1, CXCL-1 and CXCL-10, which are upregulated after viral infection or liver injury and play an important role in the induction of hepatic inflammatory responses^43–46^. MCP-1 is a potent chemoattractant for monocytes and DCs^33^, whereas CXCL-1 and CXCL-10 have been shown to recruit neutrophils, monocytes and NK cells^34,35^. Here, we confirm the production of human MCP-1, CXCL-1 and CXCL-10 by human hepatocytes engrafted in the mouse liver in hu-mice. Given the relatively low levels of amino-acid identity of these chemokines between human and mice, which are 68.4, 57.6 and 68.4% for MCP-1, CXCL-1 and CXCL-10, respectively^25^, human cells are unlikely to respond efficiently to their murine counterparts. Thus, human chemokine production by the engrafted human hepatocytes is considered one of the mechanisms improving human immune reconstitution in the liver of LTH hu-mice.

Mouse hepatocyte cell line cells have been shown to produce hematopoietic cytokines, including IL-3, GM-CSF and M-CSF^47,48^. Using human hepatocyte cell lines, previous studies demonstrated that human hepatocytes may facilitate the survival and proliferation of NK or NK-like T cells in vitro through direct cell–cell interaction and IL-15 expression^49,50^. In this study, we found that human hepatocytes engrafted in the mouse liver produce a number of important hematopoietic cytokines, including IL-3, IL-15, GM-CSF and M-CSF. IL-3, GM-CSF and M-CSF are known to be essential for the development, differentiation and proliferation of monocytes/macrophages and DCs^29,38,39^, and IL-15 is required for NK cell differentiation and proliferation^32^. As human cells do not or poorly respond to the mouse counterparts of these cytokines^25,36–39,51^, the production of these cytokines by the engrafted human hepatocytes is believed to be largely responsible for the improved human immune reconstitution in the liver of LTH hu-mice.

In summary, the present study demonstrates that engrafted human hepatocytes can significantly improve human immune reconstitution in the liver of hu-mice. Importantly, our data also indicate that the interaction of tissue-resident immune cells with hepatocytes and/or hepatocyte-derived cytokines and chemokines plays an important role in the development and maintenance of local immunity in the liver. Furthermore, the Jo2 antibody-based protocol makes it possible to achieve human hepatocyte engraftment in all strains of immunodeficient mice without the need of hepatocyte-apoptotic transgene expression, and LTH hu-mice provide a potentially useful in vivo system for modeling the immunopathogenesis and interventions of human hepatotropic virus infections such as by hepatitis B and hepatitis C virus.

## Materials and methods

### Mice and human samples

NOD-Prkdc^em26Cd52^Il2rg^em26Cd22^/Nju (NOD/SCID IL2rg^-/-^ mice or NCG) mice were purchased from Nanjing Biomedical Research Institute of Nanjing University and were housed in a specific pathogen-free (SPF) micro-isolator environment. The mice were used in experiments at 3–4 weeks of age. Discarded human fetal tissues with gestational age of 17–20 weeks and adult human liver samples from patients undergoing partial hepatectomy for primary or secondary tumors were obtained with informed consent at the First Hospital of Jilin University. Protocols involved in the use of human tissues and animals were reviewed and approved by the Institutional Review Board and Institutional Animal Care and Use Committee of the First Hospital of Jilin University (protocol no. 2012-112), and all of the experiments were performed in accordance with the protocols.

### Preparation of human tissues and cells

Human fetal thymus, fetal hepatocytes and liver-derived CD34^+^ HSPCs were isolated from human fetus with gestational age between 17 and 21 weeks as previously described^22,52^ with some modifications. In brief, to separate FHCs and CD34^+^ HSPCs, the fetal liver tissue was mechanically minced and digested with the buffer containing 0.05–0.075% of type IV collagenase (Life Technologies) and 0.005% of DNase I (Roche) at 37 °C water bath for 30–40 min. After passing through a 70 μm mesh, the cell suspension was centrifuged at 250 *g* for 5 min. The cell pellets were used to further enrich FHCs by low speed centrifuge, whereas the cell suspension was used to isolate CD34^+^ HSPCs using magnetic-activated cell sorting (MACS), CD34 Micro-Bead Kit, Miltenyi Biotec, Auburn, CA). The purities of FHCs and CD34^+^ HSPCs were both >90%, and cell viabilities evaluated by Trypan blue exclusion were routinely >95%.

### Construction of hu-mice

Hu-mice with human lymphohematopoietic reconstitution were created by co-transplantation of human fetal thymic tissues (under kidney capsule) and fetal liver-derived CD34^+^ HSPCs (1.5–2 × 10^5^/each, i.v.) into sublethally (1.75 Gy)-irradiated NCG mice (these Thymus/HSPC-grafted mice are referred to as TH hu-mice hereafter for the sake of simplicity), as previously described^22^. To construct liver-chimeric TH hu-mice (referred to as LTH hu-mice), NCG mice received intrasplenic injection of FHCs (1–2 × 10^6^/mouse)^53^ 3 weeks prior to sublethal total body irradiation and transplantation of human fetal thymus and CD34^+^ HSPCs. To facilitate human hepatocyte engraftment and proliferation, mice were intraperitoneally administrated with anti-mouse Fas antibody (Jo2; BD) to induce mouse hepatocyte apoptosis at the dose and schedule wherein described. Liver injury following Jo2 treatment was evaluated by measuring serum levels of alanine aminotransferase using a diagnostic kit (Jian Cheng, Nan Jing, China) according to the manufacturer’s instruction.

### Histology

Tissues were harvested and fixed with 10% of formalin overnight, and embedded in paraffin. Serial sections (4 μm) were prepared and analyzed for H&E and immunohistochemistry (IHC). For IHC, tissue sections were stained with monoclonal mouse anti-human hepatocyte-specific antigen (Hep Par1, clone OCH1E5; DAKO), CK8/18 (clone 5D3; Mai Xin), CD45 (clone 2B11^+^PD7/26; DAKO), CD68 (clone PGM1; DAKO), CD20 (clone L26; DAKO), monoclonal rabbit anti-human CD11c (clone EP1347Y; Abcam) or polyclonal rabbit anti-human CD94 (BOSTER), and the immunoreactivity was detected with UltraSensitive^TM^ Streptavidin-Peroxidase Kit (KIT-9710, Mai Xin, China) according to the manufacturer’s protocol. The numbers of positively stained cells per field were assessed using the Image J (NIH), and at least seven randomly selected fields per tissue section were analyzed.

### Multiplexed IHC staining

Multiplexed IHC staining was performed on paraffin-embedded liver sections to determine special association between human hepatocytes and immune cells using Opal^TM^ Multi-color IHC kit (PerkinElmer, Waltham, MA) according to the manufacturer’s instruction. Briefly, tissue sections were dewaxed and rehydrated, and antigen retrieval and quenching endogenous peroxidase activity were performed with AR6 buffer using microwave treatment. The slides were washed and blocked with the PerkinElmer Antibody Diluent/Block buffer, followed by primary antibody staining. Then, the slides were washed and incubated in the Opal polymer HRP Ms + Rb for 10 min, and visualized by tyramide signal amplification dye. After that, the slides were microwave treated with the AR6 buffer for removing the antibodies in order to introduce the next primary antibody. When all antibody staining is completed, the slides were 4,6-diamidino-2-phenylindole (DAPI) stained and cover slipped with Prolong Gold Antifade Reagent (Thermo Fisher). In addition, single color control staining with each primary antibody was also prepared. The primary antibodies used are detailed in Table S1. The slides were imaged and processed using the Zeiss LSM880 Confocal Microscope. Images were analyzed and quantified with Image J (NIH).

### Enzyme-linked immunosorbent assay (ELISA)

Serum levels of human albumin were measured using a human albumin ELISA quantitation kit (Bethyl Laboratories) according to the manufacturer’s protocol.

### Quantitative RT-PCR

Total RNA was extracted from liver tissues by Trizol and complementary DNA (cDNA) was synthesized using a cDNA Reverse Transcription kit (code no. RR047A, TAKARA). Quantitative PCR was performed using the SYBR Green PCR mix (code no. RR820L, TAKARA) on a PRISM 7700 (Applied Biosystems). The specificity of the primers used (Table S2) was previously confirmed^54,55^.

### Co-culture of human immune cells with human hepatocytes

FHCs were plated into rat-tail collagen-coated 24-well plate at 2 × 10^5^ per well in primary hepatocyte maintenance medium (PHM medium) (CM4000; Thermo Fisher Scientific), 24 h later, the unattached FHCs were washed out and autologous CD45^+^ cells (purified from the CD34^–^ fraction of FLCs) were seeded (5 × 10^4^ per well). CD45^+^ cells cultured in the PHM medium without human hepatocytes were used as controls. A half medium change was performed twice a week.

### Flow cytometry

The phenotype and composition of human immune cells were measured by multi-color flow cytometric (FCM) using various combinations of the following mAbs: anti-human CD45, CD19, CD3, CD4, CD8, CD14, CD33, NKp46, anti-mouse CD45, Ter119 (anti-human CD33 was obtained from Biolegend; the rest antibodies were purchased from BD PharMingen, San Diego, CA). FHCs were fixed and permeabilized with BD Cytofix/Cytoperm Kit, followed by staining with polyclonal rabbit anti-human albumin/FITC (DAKO) or polyclonal rabbit IgG isotype control/FITC (Thermo fisher) antibodies. FCM analysis was performed on a FACS Fortessa (BD Biosciences). Dead cells were excluded from the analysis by gating out lower forward scatter and high propidium iodide-retaining cells.

### Statistical analysis

The level of significant differences in group means was determined by the Student’s *t*-test. All statistical analysis was performed using Prism 7 (GraphPad Software). A *p-*value of ≤ .05 was considered significant in all analyses herein.

## Electronic supplementary material

Supplementary Figures and Tables
