# Supplementary material for: Humanized mice reveal an essential role for human hepatocytes in the development of the liver immune system
Source: Cell Death Dis. 2018 Jun 4;9(6):667. doi: 10.1038/s41419-018-0720-9 (PMC5986801; doi:10.1038/s41419-018-0720-9)
Supplement: Supplementary file 1 — Supplementary Figures and Tables [file 41419_2018_720_MOESM1_ESM.docx]

**Humanized mice reveal an essential role for human hepatocytes in the development of the liver immune system**

Jinglong Guo^a,b,c^, Yang Li^a,b,c,d^, Yanhong Shan^a^, Chang Shu^a^, Feng Wang^a,b,c^, Xue Wang^a,b,c^, Ge Zheng^e^, Jin He^a^, Zheng Hu^a,b,c,1^, Yong-Guang Yang^a,b,c,d,1^

**Supplementary Information**

Supplementary Figures: 6

Supplementary Tables: 2

**
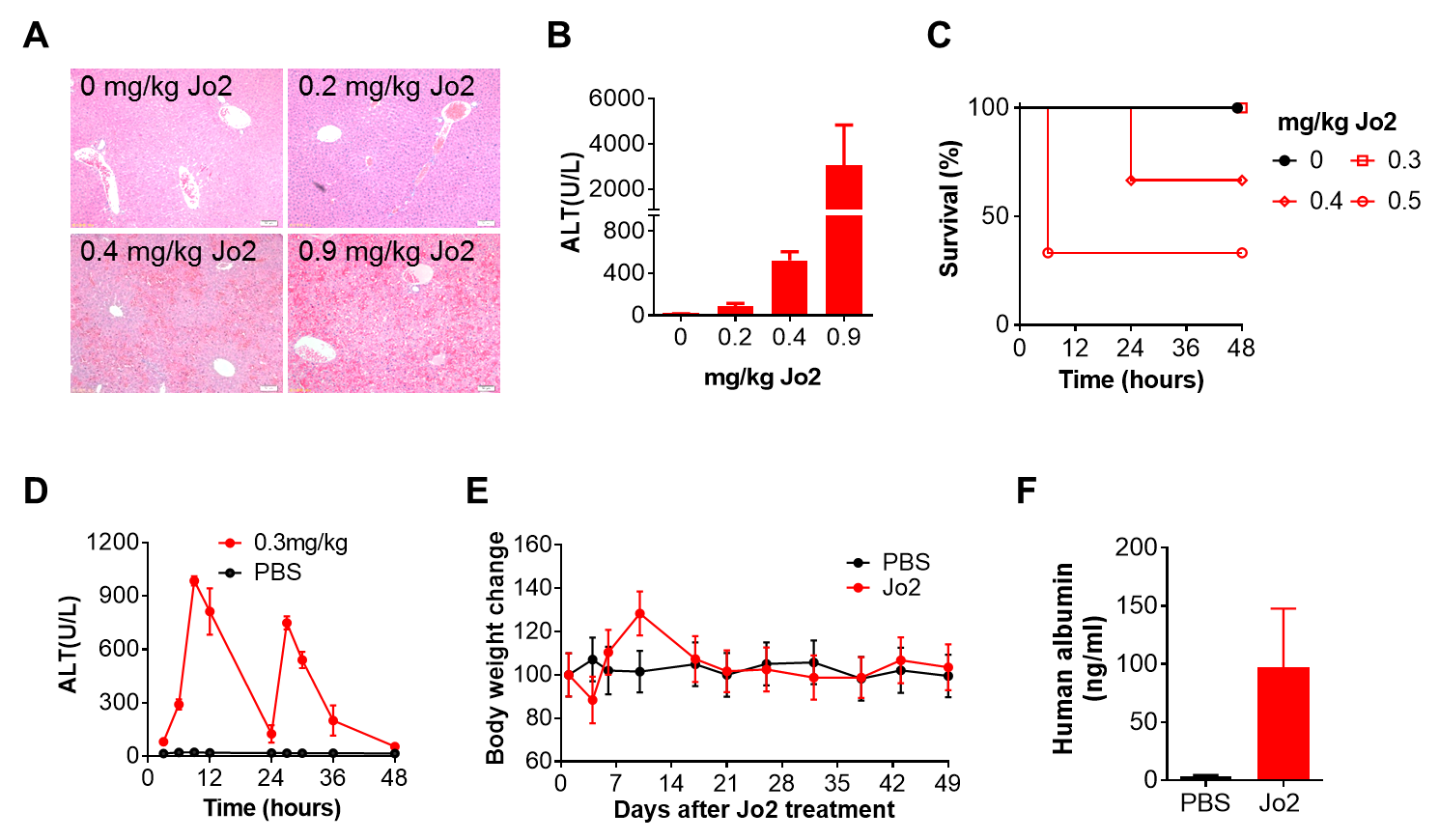
**

**Figure S1. Optimization of Jo2 antibody dose and schedule for inducing mouse liver injury and facilitating human hepatocyte engraftment.** (A, B) Representative H&E staining profiles of liver tissue sections (A) and serum ALT levels (B) from NCG mice 3 hours after injection (i.p.) of Jo2 antibody at the indicated doses (n=3 per group). (C) Survival of NCG mice treated with Jo2 antibody at the indicated doses (n=3 per group). (D) The serum ALT levels in NCG mice treated with Jo2 antibody (0.3mg/kg per injection twice with a 24-hour interval; n=3) or injected with PBS (n=3) at the indicated times following first injection of Jo2 antibody. (E) Bodyweight changes of NCG mice that were treated with Jo2 antibody (0.3mg/kg every other day; n=3 per group) or PBS (n=3) for 7 weeks. (F) NCG mice were intrasplenically transplanted with human fetal hepatocytes (FHCs; 1.8×10^6^/mouse), followed next day by treatment with Jo2 antibody (0.3mg/kg; n=5) or PBS (n=3) every 3 days for 3 weeks. Shown are serum levels of human albumin at week 3 post-FHC transplantation.

**
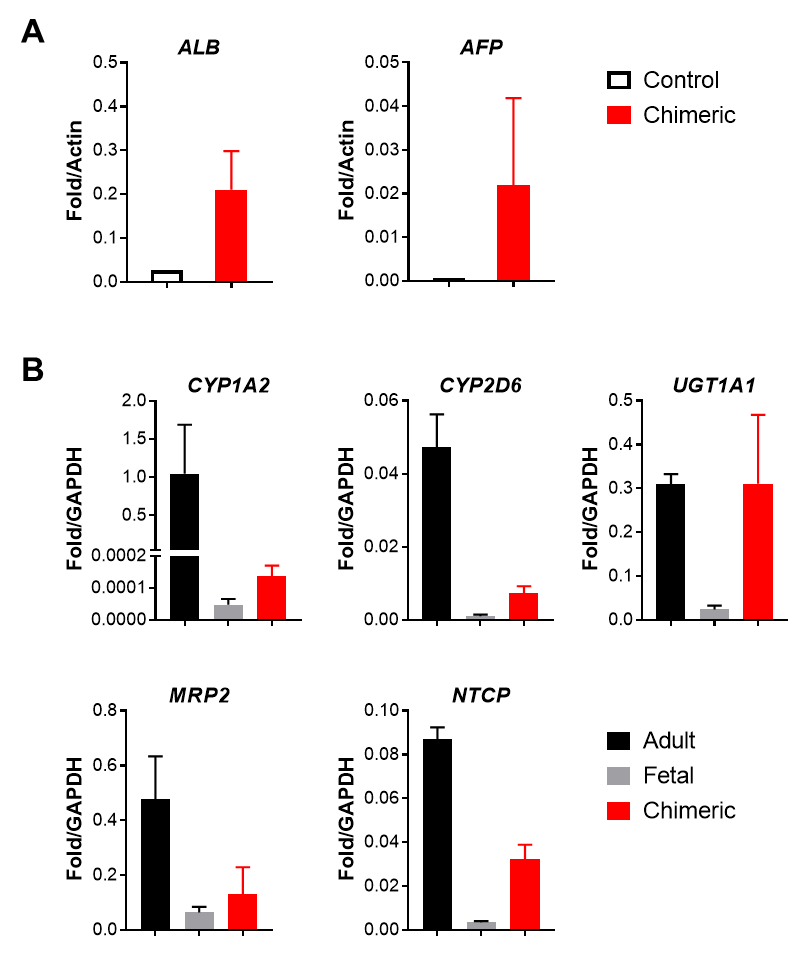
**

**Figure S2. Human hepatocyte specific gene expression in the livers of human hepatocyte-grafted mice.** NCG mice received intrasplenic transplantation of human FHCs, followed next day by Jo2 antibody treatment (0.3mg/kg per injection every 3 days; i.p.) for 10 weeks. Liver tissues were prepared from these mice 14 weeks after FHC transplantation and analyzed for human hepatocyte-specific gene expression by qRT-PCR. Mouse liver samples from non-transplanted NCG mice, adult or fetal human liver tissues were used as controls. (A) Expression levels of human hepatocyte-specific genes (*ALB* and *AFP*) in the liver tissues from human FHC-transplanted NCG mice (Chimeric, n=3) and control NCG mice (Control). Values were normalized to total (mouse plus human) *ACTIN*. (B) Expression levels (mean ± SEMs; normalized to human *GAPDH*) of human hepatocyte metabolic genes, phase I enzymes *CYP1A2* and *CYP2D6*, phase II enzyme *UGT1A1*, and transporters *MRP2* and *NTCP* in human adult livers (n=3), human fetal livers (n=3), and the liver tissues from FHC-transplanted NCG mice (Chimeric Liver; n=3).


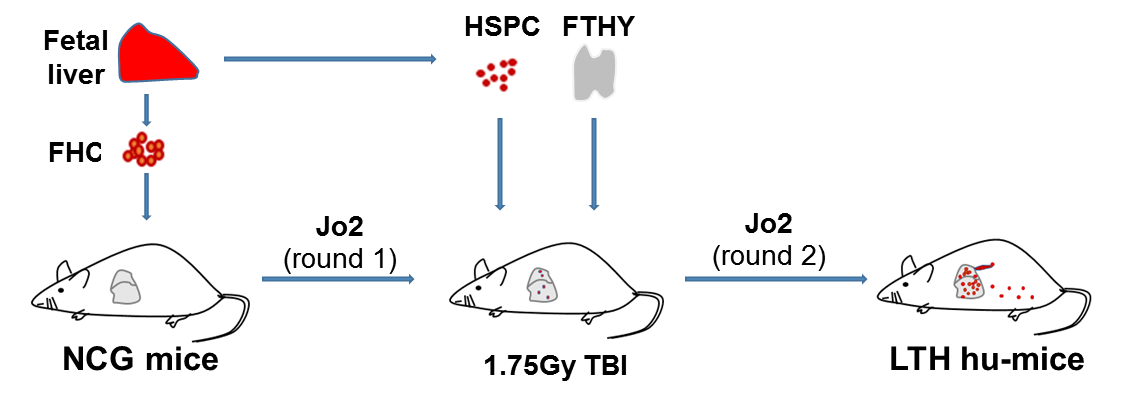


**Figure S3. Schematic over view of LTH hu-mouse construction.** NCG mice were intrasplenically transplanted with human FHCs and 3 weeks later, received sublethal total body irradiation (TBI) followed within a day by transplantation of human FTHY (under renal capsule) and CD34^+^ HSPCs (i.v.). To promote human hepatocyte engraftment and proliferation, mouse liver injury was induced by Jo2 antibody treatment (0.3mg/kg/injection every 3 days; i.p.) for 2 rounds, which were given from day 1 for 2 weeks (5 injections) and from week 3 to week 7 (7 injections), respectively.


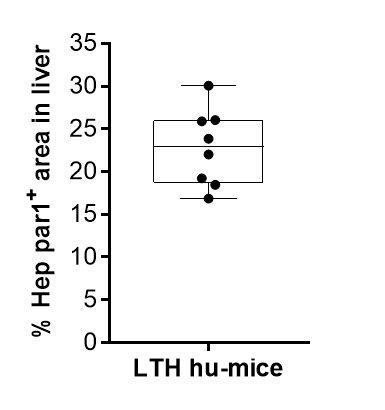


**Figure S4. Levels of human hepatocyte repopulation in the livers of LTH hu-mice.** Sections of liver tissues from LTH hu-mice at approximately 15 weeks after transplantation of human FTHY/CD34^+^ HSPCs (n=8) were stained with anti-human Hep par1 antibody. Each symbol represents the average Hep par1^+^ area (%) in the liver from each individual LTH mouse (5-9 random areas per sample were analyzed by Image J).


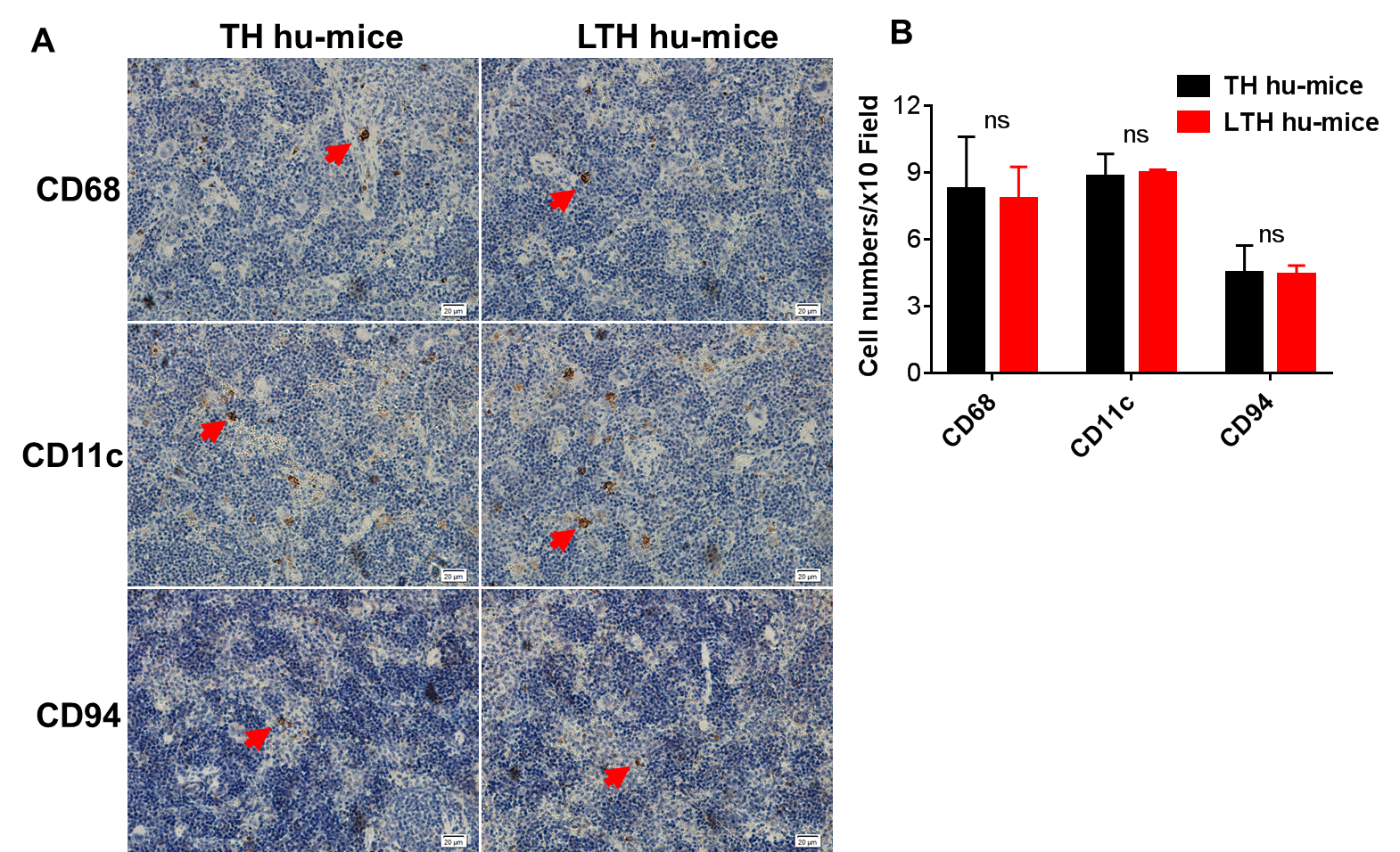


**Figure S5. Human immune cell reconstitution in the spleens of TH and LTH hu-mice**. Spleen tissue sections from TH and LTH hu-mice at approximately 15 weeks after transplantation of human FTHY/CD34^+^ HSPCs (n=3 per group) were stained with anti-human CD68, CD11c, and CD94 antibodies. (A) Representative IHC staining images (scale bar, 20 μm). (B) Numbers of positively-stained cells per field (at the original magnification×10; 4 random fields per sample were counted). Data are shown as Mean±SEMs. ns, not significant.


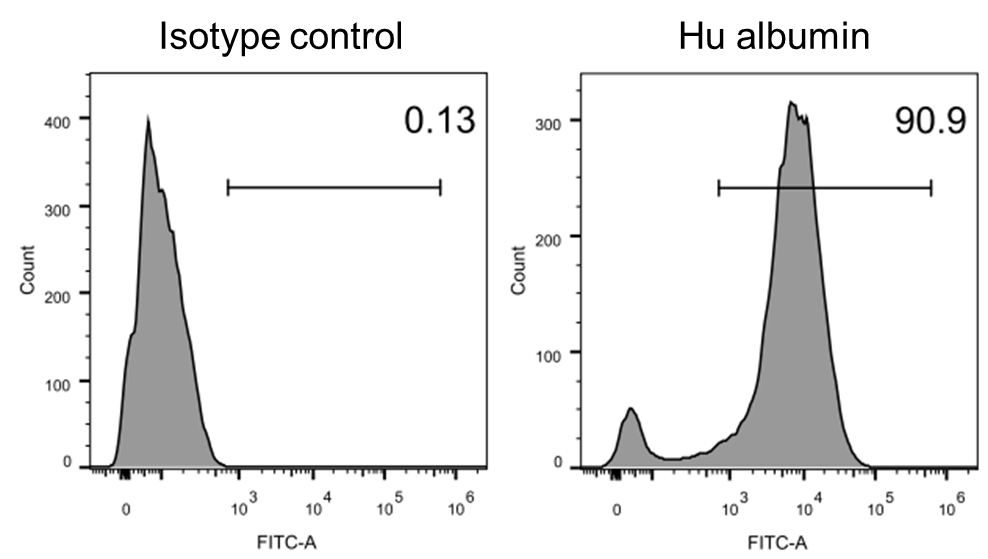


**Figure S6. The purity of human fetal hepatocytes.** The human fetal hepatocytes (5×10^5^/tube) were fixed and permeabilized, followed by staining with FITC-conjugated rabbit IgG isotype control (Left) or rabbit anti-human albumin (Right), and analyzed by FCM.

| **Table S1. Antibodies used for multiplexed IHC staining** | | | | | |
| --- | --- | --- | --- | --- | --- |
|  | | | | | |
| **Order** | **Antibody** | **Host (clone); company** | **Dilution** | **Incubation/temp** | **TSA dyes** |
| 1 | Hep par1 | Mouse (OCH1E5); DAKO | 1:100 | Overnight/4℃ | 690 |
| 2 | CD11c | Rabbit (EP1347Y); Abcam | 1:100 | 60 min/RT* | 570 |
| 3 | CD94 | Rabbit; BOSTER | 1:100 | 60 min/RT | 620 |
| 4 | CD68 | Mouse (PGM1); DAKO | 1:100 | 60 min/RT | 520 |
| 5 | DAPI | Perkin Elmer Opal 7-color kit | 2 drops/ml | 5 min/RT |  |
| * RT: room temperature | | | | | |

| **Table S2. Primers used for qPCR** | | |
| --- | --- | --- |
| **Genes** | **Forward (5^’^-3’)** | **Reverse (5’-3’)** |
| ***ALB*** | GGAATGCTGCCATGGAGATCTGC | CCTTCAGTTTACTGGAGATCG |
| ***AFP*** | CCTACAATTCTTCTTTGGGCT | AGTAACAGTTATGGCTTGGA |
| ***GAPDH*** | ACCCAGAAGACTGTGGATGG | TCTAGACGGCAGGTCAGGTC |
| ***NTCP*** | AGGGGGACATGAACCTCAG | AGGTCCCCATCATAGATCCC |
| ***CYP1A2*** | GCTTCTACATCCCCAAGAAAT | TCCCACTTGGCCAGGACT |
| ***CYP2D6*** | GCTTCGACCAGTTGCGGC | AGCCCATTGAGCACGACC |
| ***MRP2*** | CAAACTCTATCTTGCTAAGCAGG | TGAGTACAAGGGCCAGCTCTA |
| ***UGT1A1*** | CCCATGCTGGGAAGATACTGTT | GCGTCAGGTGCTAGGACAAC |
| ***MCP-1*** | CCCCAGTCACCTGCTGTTAT | TGGAATCCTGAACCCACTTC |
| ***CXCL-1*** | AGTGGCACTGCTGCTCCT | TGGATGTTCTTGGGGTGAAT |
| ***CXCL-10*** | CAATGATCTCAACACGTGGG | GTCTGAGTGGGACTAAGGG |
| ***IL-3*** | GGACTTCAACAACCTCAATGGG | TTGAATGCCTCCAGGTTTGG |
| ***IL-15*** | Product No. DHS231349, XY biotech, Shanghai, China | |
| ***GM-CSF*** | CACTGCTGCTGAGATGAATGAAA | GTCTGTAGGCAGGTCGGCTC |
| ***M-CSF*** | Product No. DHS625792, XY biotech, Shanghai, China | |
| ***ACTIN**** | TTCAACACCCCAGCCATG | CCTCGTAGATGGGCACAGT |
| * Shared by human and mouse Actin | | |
